# Prebiotic and Probiotic Modulation of the Microbiota–Gut–Brain Axis in Depression

**DOI:** 10.3390/nu15081880

**Published:** 2023-04-13

**Authors:** Daniel E. Radford-Smith, Daniel C. Anthony

**Affiliations:** 1 Department of Pharmacology, University of Oxford, Mansfield Road, Oxford OX1 3QT, UK; 2 Department of Chemistry, University of Oxford, Mansfield Road, Oxford OX1 3TA, UK; 3 Department of Psychiatry, University of Oxford, Warneford Hospital, Warneford Lane, Oxford OX3 7JX, UK

**Keywords:** gut microbiota, microbial metabolites, depression, metabolic disease, obesity

## Abstract

Emerging evidence demonstrates that alterations to the gut microbiota can affect mood, suggesting that the microbiota–gut–brain (MGB) axis contributes to the pathogenesis of depression. Many of these pathways overlap with the way in which the gut microbiota are thought to contribute to metabolic disease progression and obesity. In rodents, prebiotics and probiotics have been shown to modulate the composition and function of the gut microbiota. Together with germ-free rodent models, probiotics have provided compelling evidence for a causal relationship between microbes, microbial metabolites, and altered neurochemical signalling and inflammatory pathways in the brain. In humans, probiotic supplementation has demonstrated modest antidepressant effects in individuals with depressive symptoms, though more studies in clinically relevant populations are needed. This review critically discusses the role of the MGB axis in depression pathophysiology, integrating preclinical and clinical evidence, as well as the putative routes of communication between the microbiota–gut interface and the brain. A critical overview of the current approaches to investigating microbiome changes in depression is provided. To effectively translate preclinical breakthroughs in MGB axis research into novel therapies, rigorous placebo-controlled trials alongside a mechanistic and biochemical understanding of prebiotic and probiotic action are required from future research.

## 1. Introduction

Research on the microbiota–gut–brain (MGB) axis has increased exponentially over the past decade, exposing the far-reaching influence of the gut microbiome in health and disease [1]. The gut microbiome has become a target for novel therapeutics that aim to modify gut microbial metabolism for the benefit of host immunity, energy balance, and mental health. Prebiotics and probiotics have become a mainstay of microbiota–gut–brain axis research, both as a tool to manipulate the host microbiota and as a potential therapeutic for mood disorders including major depressive disorder (MDD) [2]. Probiotics are defined as bacteria that elicit beneficial effects on host physiology when ingested [2]. Prebiotics are substrates (typically fibre, carbohydrates, or saccharides indigestible by the host) that also confer a positive health effect when metabolised by commensal microbes, but do not include the microbes themselves [3].

Depression is common, debilitating, and lacks effective treatment options for the majority of those affected [4]. Conventional antidepressants, while very effective in some people, have not reduced the prevalence of mood disorders in the last few decades, and this is despite a massive increase in the availability and prescription of these medicines at the level of primary care, particularly in the UK [5]. NHS data have shown that the prescription of antidepressants is doubling every 10 years. In 1998 there were about 18 million prescriptions, 36 million in 2008 [6], and the latest data from 2018 showed 71 million prescriptions being given out [7]. This increase can be partly attributed due to longer treatment regimens and an improvement in awareness, identification, and treatment of mental illness in the community over the last few decades. Over a similar period (1993–present), however, the prevalence of common mental disorders has remained comparatively stable [8], with between 15 and 20% of adults (and more women than men) experiencing a common mental disorder, for the most part anxiety or depression, within the last week at the time of polling. Because current antidepressant and anxiolytic agents are not necessarily reducing the prevalence of mental illness in society, adjunct therapies should continue to be investigated. Herein, we review and discuss the efficacy, safety, and potential mechanisms by which prebiotics and probiotics may exert beneficial effects on brain function to mitigate MDD risk.

## 2. The Gut Microbiome

Comprehending the significance of very large (or very small) numbers is difficult, despite being inherent to descriptions of the biological and physical world. The human microbiome tends to be introduced with massive orders of magnitude, for example the trillions of microbes known to inhabit the digestive tract. These quantifications gloss over the fact that our interpretation of the microbiome remains limited by our own understanding and means of characterising it. The human microbiome is mostly bacterial, but also incorporates commensal residing viruses, archaea, and fungi, the functional significance of which is much less understood. On average, there are 10 commensal bacteria for every nucleated cell in the human body [9], the vast majority of which are concentrated in the colon (large intestine). The modulation and impact of colonic ‘gut’ microbes on brain health is therefore the focus of this review.

Commensal gut microbes are essential to human health. Most prominently, they have broad metabolic functions, are involved in the education and development of host immunity, and protect against the seeding of pathogenic enteric microbes [10]. Through these intimate relationships with host physiology, research on the gut microbiome has permeated all aspects of biomedical science. Initial characterisations of the human microbiome found it to be highly variable between individuals [11]. Moreover, microbiota composition has not been found to reliably predict healthy or disease-associated phenotypes [11,12], despite a shift in microbiome composition compared to healthy controls being associated with obesity and other metabolic disorders, autoimmune disorders, and psychiatric diseases. This is because associations do not distinguish meaningful differences from meaningless ones, and there is no distinction between cause and effect. This uncertainty remains a challenge of microbiome research in health and disease. This somewhat humbling revelation has led to large-scale, ‘multi-omic’ longitudinal studies to robustly characterise strain-specific microbiota profiles in healthy and disease states, with a focus on functional immune and metabolic outcomes [13,14,15,16,17]. However, these studies are still in their infancy. Further investigations that aim to characterise microbial function and specific contributions to host physiology are critical to convert basic research into clinical applications. Effort is also required to achieve improved socioeconomic and ethnic diversity of humans participating in microbiome research, so that new findings can be applied to underrepresented populations with distinct microbiome signatures [18,19].

## 3. The Microbiota–Gut–Brain Axis: Putative Mechanisms of Bug-to-Brain Communications

One of the most exciting and rapidly evolving fields of neuroscience and biological psychiatry is the MGB axis, which explores the mutual influences and communications between the gut, its commensal microbes, and brain function. Humans have never existed without a symbiotic relationship with microbes. By extension, the brain has never been without signals from the gut and its resident microbes. While much remains to be understood about how microbiota influence the brain and systemic physiology, significant progress in the last decade has provided some insight into the functional role that microbiota play in brain health and disease [1].

The MGB axis has been implicated in the pathogenesis of a wide range of neurological and psychiatric disorders. Dysbiosis of the gut microbiota, which refers to an imbalance in the composition or function of the microbiota, has been associated with various neurological and psychiatric disorders, including autism spectrum disorder, depression, and anxiety [1].

**Figure 1:**
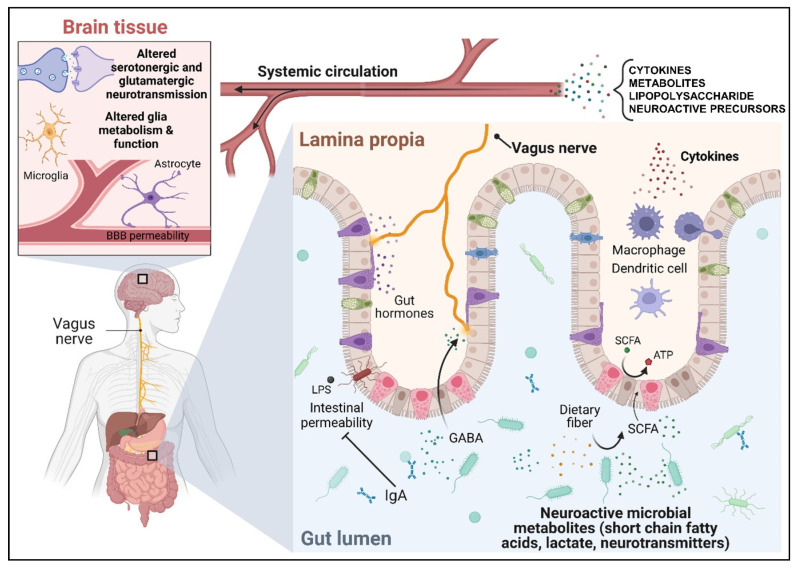
Summary of the putative mechanisms of communication between the gut microbiota and the brain.

In addition, important work in germ-free mice established the important role that the gut microbiota play to support the development of normal brain function and behaviour [20,21,22,23,24,25]. Through this research, several bidirectional routes of communication have been identified (Figure 1). Principally, these include interaction with the nervous system, in particular the vagus nerve afferents, modulation of the host immune system, and the metabolism and production of small molecules including SCFAs and neuroactive compounds. The following subsections provide a detailed overview of the MGB axis with respect to each specific pathway of communication. Most likely, these pathways operate in parallel.

### 3.1. The Microbiota–Gut–Brain Axis Communicates through the Vagus Nerve

The vagus nerve, the tenth cranial nerve, is the chief neural component of the brain–gut axis, and the primary parasympathetic nerve linking the brain with the viscera. Composed of 80% afferent (and 20% efferent) fibres, vagal afferent endings can respond to both distension of the gastrointestinal (GI) tract via mechanosensors as well as gut peptide hormones, neurotransmitters, and microbial metabolites through a diverse array of chemosensitive and other receptors [26].

Research utilising vagotomies has contributed most to our understanding of how the vagus nerve contributes to brain–gut communication, including signals from gut bacteria. In humans, vagotomy was a consequence of gastrectomy which was, historically, a treatment for duodenal ulcers. In a historical study of 118 patients following partial gastrectomy, a high incidence (43%) of psychiatric symptoms was observed [27]. Complete vagotomy has also been used for the treatment of morbid obesity [28], whereby the main cognitive symptom reported post-operatively was unexplained fatigue alongside altered hedonic perceptions of taste [29]. Reciprocally, vagus nerve stimulation has been used as an adjunct treatment for severe depression [30].

In rodents, intraperitoneal injection of lipopolysaccharide (LPS) is used to model sickness behaviour associated with peripheral and central inflammatory responses. LPS is an integral structural component of the outer membrane of Gram-negative bacteria and a potent activator of the human immune system. Several studies noted that vagotomy reduced the effect of LPS on facets of sickness behaviour, including general activity two hours post-injection [31], social behaviour [32], and food-motivated behaviour [33]. Levels of peripheral IL-1B, increased after LPS treatment, were unaffected by vagotomy [31,32]. Cumulatively, these studies point to the contribution of the vagus nerve to changes in behaviour arising from peritoneal inflammation. Conversely, vagotomy does not prevent an LPS-induced increase in brain IL-1B in rats [34]. While sickness behaviour was not measured, the results indicate that the vagus nerve is not solely responsible for communicating gastrointestinal perturbations to the brain.

Studies conducted more recently have suggested that the vagus nerve may also mediate the effects of gut bacteria on brain function and behaviour. Oral gavage treatment with *Lactobacillus rhamnosus* (JB-1) reduced anxiety and depressive-like behaviour in the elevated plus maze (EPM) and forced swim test (FST) in healthy mice, respectively [35]. These changes were associated with altered messenger (m)RNA levels of GABAA and GABAB receptors in the brain. Vagotomy prevented the behavioural effects of *L. rhamnosus* (JB-1) administration as well as the associated changes in GABAA expression in the amygdala and hippocampus. This implicates the vagus nerve in some of the observed molecular and behavioural effects of the probiotic strain. Similarly, it was shown that anxiety-like behaviour associated with dextran sodium sulphate-induced colitis was mitigated by Bifidobacterium longum (NCC3001), but only in animals with an intact vagus nerve [36]. Lastly, the bacterial species *Lactobacillus reuteri* was shown to act specifically via the vagus nerve in its ability to restore normal social behaviour in mouse models of autism spectrum disorder (ASD) when administered in drinking water [37]. This was thought to be independent of other commensal microbes, as *L. reuteri* was also able to rescue aberrant social behaviour in germ-free (GF) mice, and supplementing ASD-like mice with *L. reuteri* did not change the overall gut microbiota composition of the animals. Despite the rigorous nature of this study in elucidating the basic mechanism of action of *L. reuteri*, it remains unclear from all these studies how microbes interact with the vagus nerve. This is an important issue to resolve, as the latter study described here suggests that *L. reuteri* has the capacity to rescue social behaviour and restore oxytocin levels in the paraventricular nucleus of the hypothalamus as one strain among hundreds, at least. Understanding and harnessing this mechanism will be important in translating and applying microbiome-based therapeutics in the future via modulation of the vagus nerve.

### 3.2. The Microbiota–Gut–Brain Axis Communicates through the Immune System

Inflammation is associated with depression in a bidirectional manner [38]. Chronic gut, liver, and brain inflammatory diseases such as inflammatory bowel disease (IBD), non-alcoholic fatty liver disease (NAFLD), and multiple sclerosis (MS) are associated with increased rates of depression [39,40,41]. The peripheral immune system, therefore, is a high plausible indirect route of communication between the collective gut microbiota, brain function, and behaviour.

Studies in GF mice established the essential role for commensal microbes in the maturation of the host innate and adaptive immune systems [42] mediated by bacterial polysaccharide A [43]. GF mice have an underdeveloped immune system, including reduced number of adaptive immune cells and reduced development of secondary lymphoid structures [44]. In the GI tract itself, converging mechanisms compartmentalise commensal microbes and prevent intrusion into sterile tissue and aberrant immune responses. A dense and insoluble luminal mucous barrier, largely formed of the mucin MUC2, is maintained by goblet cells in the epithelial lining of the large intestine. Within the mucous layer, dendritic cells sample microbial epitopes in a perpetual state of immunotolerance induced by MUC2-associated glycans [42,45]. In parallel, microbial production of butyrate induces the formation of regulatory T cells [46,47] which, together with the tolerogenic dendritic cells, promote B cell secretory IgA production to further prevent pro-inflammatory innate immune activation in the gut [48,49].

Collectively, it has become apparent that the qualitative composition of the gut is crucial to maintaining systemic immune homeostasis. It is not just the presence or absence of microbes that affect host immunity, but rather the balance of an unfathomably large ecosystem. Dysbiosis of the microbiome by diet or other means therefore has the potential to disrupt immune homeostasis. Given that up to 80% of human immune cells reside in the GI tract [50], it is conceivable that the downstream implications of disrupted immune homeostasis in the gut, such as increased circulating pro-inflammatory cytokines, induce or exacerbate chronic inflammatory diseases and psychiatric disorders [44].

In a maternal immune activation (MIA) model of ASD in mice, offspring showed increased intestinal permeability, gut microbiota dysbiosis, and increased colonic inflammation by increased IL-6 expression alongside reduced social behaviour [51]. Treatment of the offspring with Bacteroides fragilis normalised levels of GI tract inflammation and reversed aberrant social behaviour, supporting an immune-mediated link between the microbiota, gut barrier integrity, and brain and behaviour [51]. Distal to the gut, a complex microbiota has also been shown to be crucial for microglia maturation and ongoing function [52]. Researchers found that microglia from GF mice, under homeostatic “steady-state” conditions, were immature and had a diminished immune response [52]. Accordingly, microglia from GF mice failed to mount an appropriate immune response to intracranial injections of LPS or lymphocytic choriomeningitis virus, characterised by reduced mRNA expression levels of TNF and IL-1B. The precise coordinates of the intracranial injection was not reported, which limits the interpretability and reproducibility of this finding. Antibiotic depletion of the microbiota produced a similar immature microglial phenotype in adult mice, suggesting an ongoing and highly plastic relationship between the gut microbiota and brain microglia [52].

The absence of microbiota has also been associated with an altered microglia phenotype in aged mice [53]. Specific pathogen-free (SPF), but not GF mice, showed an increase in intestinal permeability at ~2 years age compared to ~2 months age. This coincided with a detrimental accumulation of the microbial metabolite N 6-carboxymethyllysine in the aged microglia, which was not found in the brain of GF mice. GF mice showed reduced markers of brain oxidative stress and microglial dysfunction compared to SPF mice, which was attributed to the N 6-carboxymethyllysine. Thus, the commensal microbiota appears to play a role in normal microglia physiology and age-related pathology throughout the lifespan.

### 3.3. Microbiota–Gut–Brain Axis Communicates through Circulating Gut-Derived Microbial Metabolites and Neurotransmitters

Metabolites produced by microbiota are thought to be the key effector molecules that allow the microbiota to communicate and influence the function of distal organs, including the brain. Using untargeted metabolomics by mass spectrometry (MS) to compare the plasma of GF and SPF mice, Wikoff et al. showed that the gut microbiome affects the blood concentrations of many metabolites [54]. Tryptophan, for example, was present at higher concentrations in GF mice, while serotonin was present at higher concentrations in SPF mice, which may suggest a role for the microbiota in the metabolism of dietary tryptophan to serotonin [54]. Untargeted metabolomics of the brain, blood, and faeces of SPF mice showed that they exhibit elevated levels of neurotransmitters in the gut compared to GF mice, including serotonin, GABA, and glutamate, among others [55]. Other microbial-derived metabolites elevated in the serum of SPF mice compared to GF animals, such as indoxyl sulphate and indole-3-lactate, were also markedly elevated in the brain. In a different study, indoxyl sulphate was one of a panel of microbial metabolites required for normal fear extinction learning in mice, a behaviour that was suggested to rely on microbiota-derived signals throughout the lifespan of the mice [56]. Together, these studies substantiate the link between microbial metabolites, brain function, and altered behaviour.

Several studies have identified one or several metabolites that, when administered to GF/antibiotic-treated mice, partially or fully reproduce the effects of the presence of the gut microbiota on aspects of host immunity, brain function, and behaviour. Microbially derived butyrate and propionate, for example, are potent histone deacetylase (HDAC) inhibitors in vitro and in vivo. In the colonic mucosa, these SCFAs have been shown to act via this mechanism to switch off dendritic cell pro-inflammatory cytokine expression and enhance Foxp3 expression to drive the induction of regulatory T cells and immune tolerance [46]. As previously discussed, ongoing signals from a complex gut microbiota are required for normal microglia function in the brain [52]. By contrast, the microglia of GF mice lack these microbial signals, and display an immature phenotype including increased expression of the survival and growth factors CSF1R and DDIT4, whereby expression is typically attenuated in mature microglia. A 4-week supplementation with a mixture of SCFAs (acetate, propionate, and butyrate) in drinking water was shown to restore normal gene expression profiles in the GF mice, including reduced DDIT4 expression, and reduce the number of CSF1R+ cells to the levels exhibited by SPF mice [52]. In a follow-up study, they identified that the primary metabolite mediating microglial fitness in SPF mice was microbially derived acetate. GF mice showed depleted levels of acetate in the brain and blood compared to SPF mice. Oral gavage of 13C-derived acetate in GF mice was shown to enter brain tissues after six hours, by which point the 13C label had been incorporated into tricarboxylic acid cycle metabolites.

Microbial metabolites can also facilitate the effects of the maternal gut microbiota on offspring brain development [57]. Embryonic brain development markedly differs between GF and SPF mothers who are otherwise healthy. Specifically, axonal projections between the thalamus and cortex fail to connect properly, leading to defects in sensorimotor behaviours. Metabolomic profiling of the maternal sera and offspring brain revealed reduced levels of certain microbial metabolites that, when supplemented to antibiotic-treated dams, restored normal axonogenesis and sensorimotor behaviour in the offspring [57]. In this way, microbial metabolites not only contribute to gut–brain communication across the organism’s lifespan, but also between mother and foetus during the perinatal period.

## 4. Perturbed Microbiota–Gut–Brain Axis Metabolism in Mood Disorders

### 4.1. Profiling Gut Microbiota Changes in Mood Disorders: The Way Forward

The gut microbiota can be profiled using several complementary omics approaches, including transcriptomics, metagenomics, metaproteomics, and metabolomics. Transcriptomics, which involves the untargeted analysis of RNA molecules in a sample, provides an insight into the gene expression patterns of the microbiota [58]. Metagenomics, on the other hand, involves the direct sequencing of microbial DNA from environmental samples, providing information on the taxonomic and functional diversity of the microbiota [59]. Metaproteomics involves the analysis of proteins expressed by the microbiota, providing information on the functional capacity of the microbial community [60]. Together with metabolomic profiling, these omics approaches can provide complementary information on the taxonomic composition, function, and metabolic capacity of the gut microbiota. Their integration can serve as a comprehensive set of tools with which to explore the complex set of interactions between the gut microbiota and the host.

Several studies in the last decade have found relationships between the gut microbiome and major depressive disorder (MDD) in humans [61,62]. Despite these significant findings, there is no consensus on which bacterial taxa, if any, are specific to depressive symptoms [61]. Interestingly, a meta-analysis of microbiome studies across a spectrum of psychiatric disorders found no real specificity between the relative abundance of different microbial taxa and a given disease, and no decrease in alpha-diversity (within-patient diversity or “richness”) [62]. Rather, the most consistent observation were similar patterns of microbial changes between each disorder compared to the healthy control group, including a reduction in the abundance of butyrate-producing bacteria in patients with MDD, bipolar disorder, schizophrenia, and anxiety [62]. A high level of inter-study variation was observed. These discrepancies are likely due in part to methodological differences between studies, but also may be due to the substantial interindividual variation in gut microbiome composition [11]. Given that functional metabolic processes are conserved across bacterial taxa [11], variable gut microbial taxonomy between depressed individuals is likely to exist, but may still give rise to highly convergent downstream functional outcomes. As a result, analysis of faecal and/or gut mucosal metabolites, or functional analysis of retrieved genomic data, may be more informative than describing the relative abundance of bacteria in the gut [63]. This concept is supported by a 2019 study investigating the inter-individual variation between the faecal microbiome and the faecal metabolome [64]. It was found that the participants (>1000 unrelated twins recruited from the UK twin registry) shared approximately double the number of metabolic pathways (82%) than microbial species (43%). Fundamentally, the study revealed that a variable microbial taxonomic signature does not necessarily lead to equivalent volatility in the downstream biochemistry. It is therefore more intuitive to explore the microbiome by using integrative, complementary approaches to microbiota profiling, for example faecal metabolomic and annotated metagenomic methods that can report on function, as opposed to the classical 16S ribosomal RNA (rRNA) amplicon sequencing, which is comparatively information-poor.

### 4.2. Animal Studies

Metabolites produced by the gut microbiota play a role in host pathways related to energy metabolism, immunity, and inflammation that connect the gut, liver, and brain [65]. Direct evidence has been provided by studies that have demonstrated neurobehavioural changes associated with faecal microbiota transplantation (FMT) from humans with MDD into rodents. In comparison to germ-free (GF) mice colonised with microbes from healthy (control) individuals, GF mice receiving microbes from patients with MDD showed increased depressive-like behaviours after 2 weeks [66]. This included a reduction in the time that the mice spent in the centre of an open field test (OFT) and increased time spent immobile in the FST. Depressive-like behaviour was linked to changes in carbohydrate and amino acid metabolism in faecal and serum samples, as well as in the hippocampus [66]. However, different metabolites within these pathways were either increased or decreased in mice receiving FMT from depressed individuals. Hippocampal glycine levels were reduced in mice who received the “depressed microbiota” compared to mice who received the “healthy microbiota”. Given that glycine is required to facilitate glutamatergic neurotransmission at N-methyl-D-aspartate receptors (NMDARs), this particular metabolic alteration may have contributed to the altered behaviours that were observed [67]. For example, hypofunction of NMDARs by alterations to the glycine binding site are thought to be implicated in the pathophysiology of schizophrenia in humans [68]. FMT in mice from depressed patients mediated the neuroimmune and neuroendocrine pathophysiology of depressive-like behaviour [69]. Depressed patients showed an increase in plasma pro-inflammatory cytokines, cortisol, and the kynurenine/tryptophan ratio. Following FMT, similar changes in plasma tryptophan metabolism were observed in recipient rats, but no changes in the level of pro-inflammatory cytokines or cortisol were observed. The rats also showed increased depressive-like behaviour. Both depressed individuals and the recipient rats showed reduced richness and diversity in the composition of the gut microbiome. From these studies, it can be concluded that microbe composition associated with depression can induce depressive-like behaviours in rodents, which may be mediated by host changes in energy metabolism and the levels of endogenous neuromodulatory metabolites. However, it remains uncertain from these studies whether microbiota that are associated with MDD in humans also directly contribute to inflammation. A more comprehensive assessment of host immunity post-FMT, for example the measurement of intestinal barrier integrity, circulating endotoxin levels, quantification of specific immune cell populations, and host response to immune challenges, is warranted.

A recent systematic review evaluated 241 metabolomic studies in animal models of depression [70]. Individual metabolites were investigated by a simple vote-counting method. In the brain, levels of serotonin, dopamine, and GABA amongst others were significantly downregulated, whereas kynurenine and myo-inositol were consistently upregulated. In studies that specifically investigated the metabolite composition of the prefrontal cortex (PFC), only glutamate was significant, which was significantly downregulated. In the blood, the most consistently upregulated metabolite in animal models of depression was N-acetyl glycoproteins. These results will have been influenced by the underlying methodologies of each study, including the analysis technique (nuclear magnetic resonance [NMR] spectroscopy or MS) and how depressive-like behaviour was induced and measured. Chronic mild stress (CMS) was the most common model of depression in the systematic review [71]. This may have influenced the prevalence of inflammation-related metabolites present as CMS has been associated with the induction of peripheral inflammation [72,73]. Additionally, MS was employed in the reviewed literature more often than NMR spectroscopy or magnetic resonance spectroscopy (MRS). While high inter-study variability was noted, trends towards decreased neurotransmitter levels in the brain, including reduced glutamate levels, and increased markers of inflammation in the brain and periphery were found to be the prominent features [70].

It is worth noting that substantially less research has focused on anxiety-like behaviours in relation to the microbiota–gut–brain axis. In humans, however, anxiety and depressive symptoms are highly comorbid [74], and in a large proportion of animal models for depression, anxiety-like behaviour is often assayed as part of a battery of behavioural tests by virtue of the OFT or EPM [66,75]. In one study [75], anxiety-like behaviour was related to the gut-derived metabolite 4-ethylphenyl sulphate (4EPS). 4-ethyl phenol is derived from dietary tyrosine via gut microbiota metabolism before sulphation to 4EPS by the host. When gnotobiotic animals were colonised with single strains that either did or did not produce 4EPS, 4EPS was shown to accumulate in the brain after injection of probenecid, which prevents small molecule efflux across the blood–brain barrier (BBB). It was therefore concluded that this gut-derived molecule was able to access the brain parenchyma. 4EPS was found to increase anxiety-like behaviour, but not cognition (Y-maze) or motor function (beam traversal, pole descent, wire hang tests) by disrupting oligodendrocyte maturation and myelination [75].

To conclude, there is substantial evidence for altered gut and brain metabolism in preclinical models of mood disorders—both in terms of the levels of endogenous neurotransmitters and metabolites that are exclusively microbially derived. Much of this evidence has accumulated recently in the last few years, and further research will be required to corroborate these findings in individuals with MDD and/or anxiety to assess the translational validity of candidate metabolites.

### 4.3. Human Studies

Clinical studies have confirmed that there is perturbation of the microbiota–gut–brain axis, including alterations to individual metabolites, in patients with mood disorders. In two independent studies, the abundance of *Alistipes* spp. has been shown to be elevated in individuals with MDD compared to controls [76,77]. Mechanistically, *Alistipes* spp. may be related to metabolic pathways associated with increased inflammation, as another study showed correlations with intensity of abdominal pain in irritable bowel syndrome [78]. Through the use of gut microbiota sequencing in tandem with gut metabolomics analysis, changes in gut microbiota composition between depressed and non-depressed individuals have been related to changes in host metabolism [79]. Bacteroides spp. were enriched in individuals with MDD and correlated with reduced faecal amino acid levels (leucine and proline) and increased lipid levels (2-indolecarboxylic acid and itaconic acid). Separately, the authors found that GABA and related metabolites were reduced in the gut in those with MDD compared to healthy controls, and the authors speculated that these findings may influence brain GABA levels in MDD. In 2022, it was also elegantly demonstrated that a significant portion of the variation in serum GABA levels (up to 22%), which were reduced in individuals with depressed bipolar disorder compared to healthy controls, could be explained by whether or not the gut microbiota of a given individual contained genes essential for GABA synthesis [80]. Moreover, GABA levels have been shown to be increased in the blood of obese individuals after faecal microbiota transplant from lean individuals, which coincided with increased insulin sensitivity [81]. These studies provide further evidence that changes to the gut microbiota can affect the level of circulating neurotransmitters. Nevertheless, it remains unclear to what extent microbially produced neurotransmitters, for example GABA, directly contribute to central neurotransmitter levels [82].

Two large (n > 1000) cohorts with microbiome metagenomic data, alongside quality of life (QoL) or depression scores, have shed further light on the possible links between gut microbial metabolism and MDD in humans [83]. In the first cohort, two metabolites were positively associated with QoL scores, including synthesis of 3,4-dihydroxyphenylacetic acid (DOPAC, a dopamine metabolite) and isovaleric acid (a SCFA). The correlation of QoL scores with DOPAC was reproduced in the second cohort. Butyrate-producing bacteria were positively associated with QoL in both cohorts [83]. Conversely, in a study of 113 children, chronic psychosocial stress showed a significant positive correlation with the faecal levels of several SCFAs, including butyrate and isovalerate [84]. Overall, these correlational studies in humans support the preclinical evidence for a causal role of the gut microbiota and associated microbial metabolites in the pathogenesis of depressive symptoms. Many discrepancies remain in terms of the directionality of these metabolites, and how the composition of a “depressed” or “healthy” microbiome should be defined.

## 5. Modulation of the Microbiota–Gut–Brain Axis by Prebiotics and Probiotics

### 5.1. Prebiotics Modulate Brain Function and Behaviour in Animal Models

In rodents, galacto-oligosaccharide (GOS) prebiotics have been shown to elevate brain BDNF in the hippocampus. BDNF expression is thought to be important for ongoing neurogenesis in the adult hippocampus [85], and its levels in human blood have been shown to be negatively correlated with depression severity [86]. BDNF protein was also shown to be elevated in the hippocampus of rats after five-week supplementation with the human milk oligosaccharide (HMO) 2′-fucosyllactose [87]. In this case, associative learning behaviour was also assessed, and rats supplemented with the HMO tended to acquire the operant conditioning task in fewer sessions than the control group [87]. Prebiotic supplementation has also been shown to modulate anxiety and depressive-like behaviour in rodents. Mice fed GOS for three weeks were shown to be less anxious than controls (drinking water only) in the LDB 24 h after LPS administration [88]. The anxiolytic effect of the prebiotic was associated with an increase in gut Bifidobacterium spp. and an attenuation of IL-1B and 5-HT2A receptor proteins in the frontal cortex after LPS administration [88]. Prebiotics have also been shown to mitigate the adverse behavioural effects of both early life stress by maternal separation [89] and chronic mild stress [90] in rodents. This included reduced anxiety and depressive-like behaviour [90] and an increased rate of spatial learning in the Morris water maze which was independent of early life stress [89]. Lastly, postnatal supplementation of rats with GOS has been shown to reduce anxiety-like behaviour in the EPM at juvenile (3-week-old), adolescent (8-week-old), and adult (4–6-month-old) timepoints [91]. Interestingly, this effect was only observed if the rats were supplemented prior to weaning, and not when supplemented in adulthood, which suggests that the anxiolytic effects of GOS supplementation in unchallenged animals may depend on the modulation of brain function during a specific neurodevelopmental window. No overall effect of postnatal prebiotic supplementation was observed on the gut microbiome in any age group, although GOS supplementation was found to modify the relationship between branched-chain amino acid levels and age in the hippocampus [91]. Overall, there is substantial preclinical evidence to support a neuromodulatory effect of prebiotics at both juvenile and adult timepoints, despite conflicting evidence in some cases. The mechanisms largely remain unclear due to inconsistent effects of prebiotics on the microbiome—some studies have reported a bifidogenic effect whereas others report no clear effects. A proportion of this variation may be due to inconsistencies in the age at which animals were treated, the length of treatment, the type of prebiotic, and the amount of prebiotic administered. Comparing the effects of prebiotics in GF and SPF animals would allow confirmation of prebiotics’ action by modification of the microbiome in vivo. This would be important, as GOSs have been shown to have microbiota-independent anti-inflammatory effects, possibly via TLR4-mediated IL-10 production [92]. While the effects of prebiotics have not been assessed in GF animals, prebiotics have been shown to be effective in gnotobiotic animals colonised with just a single strain [93].

### 5.2. Probiotics Modulate Brain Function and Behaviour in Animal Models

It is well-established that commensal bacteria contribute to host physiology, including brain function and behaviour [1]. The contributions of individual bacterial strains to the developmental and homeostatic mechanisms involving the gut–brain axis in gnotobiotic models has been discussed in Section 3. Here, studies involving probiotic supplementation as a potential therapeutic will be discussed (Table 1).

In BALB/c mice, which exhibit an innately anxious phenotype relative to other mouse strains [94], Bifidobacterium longum 1714 reduced anxiety-like behaviour in the OFT and reduced depressive-like behaviour in the FST, while Bifidobacterium breve 1205 reduced anxiety-like behaviour in the EPM [95]. Both probiotics were administered daily for three weeks prior to the start of behavioural testing as 1 × 10^9^ colony forming units (CFU)/mL by oral gavage. In each case, a three-week treatment with escitalopram (daily oral gavage 20 mg/kg) in the positive control group did not alter the behaviour in each of these tests, relative to phosphate-buffered saline controls. It is possible that, as the BALB/c mice were healthy, the behavioural assays used may not have been sensitive enough to detect subtle changes in behaviour when the mice were unchallenged. Escitalopram treatment did reduce stereotypic behaviour in the marble burying test (MBT), as did both Bifidobacterium probiotics. While no insight was provided into how the probiotics elicited these behavioural changes, and why the behavioural changes appeared to be strain-dependent, a previous study in BALB/c mice using the probiotic *L. rhamnosus* (JB-1) showed that behavioural effects were dependent on the vagus nerve [35].

Since these early studies that pioneered the field of probiotics as novel therapeutics for mood disorders, further efforts have been made to understand how certain strains exert their effects via the microbiota–gut–brain axis. In a brain in vivo MRS study, BALB/c mice were treated with *L. rhamnosus* (JB-1) daily for four weeks, and brain imaging was performed weekly [96]. A significant increase in brain glutamate + glutamine (Glx) levels was identified after two weeks of treatment. Brain Glx remained elevated for 4 weeks after cessation of probiotic treatment. In contrast, brain NAA and GABA were elevated after four weeks of probiotic supplementation but returned to baseline levels immediately after treatment cessation. The study suggests that central levels of Glx levels may be of relevance to the enduring effects of *L. rhamnosus* (JB-1) on behaviour in mice, though the effects of probiotic supplementation on levels of glutamate and glutamine need to be individually resolved and quantified in future work.

**Table 1:** Summary of probiotic studies in rodent models on anxiety and depressive-like behaviours and brain metabolism. ↑ increase, ↓ decrease.

Model Organism (Paradigm)	Probiotic (Dose)	Duration of Treatment	Behavioural Effects	Neurochemical Effects	Reference
7-week-old male BALB/c mice (healthy)	*B. longum 1714* or *B. breve 1205* (1 × 10^9^ CFU, daily)	3 weeks prior to start of behavioural tests, 3 weeks during behavioural testing (6 weeks total)	↓ anxiety (OFT, MBT) and ↓ depressive-like behaviour (FST) (*B. longum 1714*) ↓ anxiety (EPM and MBT) (*B. breve 1205*)	Not applicable	[95]
6-week-old male BALB/c mice (vagotomy)	*L. rhamnosus JB-1* (1 × 10^9^ CFU, daily)	4 weeks (behavioural assays started during final week of treatment)	↓ anxiety (EPM, OFT) ↓ depressive-like behaviour (FST)	Altered hippocampal *GABA_A_* receptor subunit expression, effects dependent on vagus nerve	[35]
4-week-old male BALB/c mice (healthy MRI and MRS brain imaging)	*L. rhamnosus JB-1* (1 × 10^9^ CFU, daily)	4 weeks (imaging performed throughout duration of treatment at weeks 0, 1, 2, 3, 4, and 4 weeks post-treatment cessation)	Not applicable	↑ brain Glx at weeks 2, 3, 4, 8 ↑ brain NAA at weeks 1, 2, 3, 4 ↑ brain GABA at week 4 only	[96]
Male Swiss albino mice 25–30 g (28 days CMS)	*L. plantarum MTCC 9510* (2 × 10^10^ CFU, daily)	4 weeks (duration of CMS paradigm)	CMS ↓ locomotion (OFT), ↑ depressive-like behaviour (FST, TST), reversed by *L. plantarum MTCC 9510*	CMS ↑ BBB permeability, ↑ brain NF-kB, reversed by *L. plantarum MTCC 9510*	[97]
Male neonatal C57Bl/6 mice (maternal separation)	*B. pseudocatenulatum CECT 7765* (1 × 10^8^ CFU, daily)	20 days (PND2 to PND21)	MS ↑ anxiety (EPM). *B. pseudocatenulatum CECT 7765* ↓ anxiety (EPM)	Not applicable	[98]
4-week-old male Sprague Dawley rats (chronic HFD, 60% of kilocalories from fat)	*B. bifidum* W23, *B. lactis* W52, *L. acidophilus* W37, *L. brevis* W63, *L. casei* W56, *L. salivarius* W24, *Lc. Lactis* W19, *Lc. Lactis* W58 (3.75 × 10^8^ CFU/mL in drinking water, daily)	5 weeks prior to start of behavioural tests, 10 weeks total	Probiotic mixture ↓ depressive-like behaviour (FST) irrespective of diet.	Not applicable	[99]

Probiotics have also been shown to exert remedial effects in stressed rodents. In one study [97], male Swiss albino mice were subject to 28 days of CMS or left undisturbed. Half of the CMS group and half of the control group received Lactobacillus plantarum MTCC 9510 by oral gavage during the same period. Chronic treatment with L. plantarum MTCC 9510 normalised locomotor activity in the OFT, which had been significantly reduced by CMS [97]. Immobility in the FST and tail suspension test were also reduced by the CMS/probiotic compared to CMS/vehicle, but it was not discussed as to whether activity levels in the preceding OFT may have confounded these results. CMS increased serum TNF and brain NF-kB, which were prevented by the probiotic. Increased peripheral inflammation may have occurred through increased intestinal permeability and increased circulating endotoxin levels after CMS, which were also attenuated by L. plantarum MTCC 9510 treatment [97]. BBB integrity was also reduced by CMS and increased by the probiotic. Interestingly, the microbial metabolite butyrate has been shown to increase both gut barrier integrity [100] and BBB integrity [101], possibly via increased HIF-1 expression [100]. In this study, caecal butyrate was significantly increased in the CMS/probiotic mice relative to the CMS/vehicle mice, although probiotic treatment alone did not significantly increase butyrate levels. Overall, this study suggests that chronic probiotic treatment can attenuate stress-induced inflammation and depressive-like behaviours in mice by increasing intestinal and brain barrier integrity.

In a maternal separation model of early life stress, male offspring were treated with Bifidobacterium pseudocatenulatum CECT 7765 or vehicle between postnatal day (PND)2 and PND21 [98]. In the small intestine, maternal separation led to increased interferon gamma and interleukin (IL)-10, suggesting the induction of a Th1-type inflammatory response—interferon gamma being the archetypal Th1 cytokine [102]. Increased IL-10 may represent the homeostatic response to attenuate the pro-inflammatory response to early life stress. *B. pseudocatenulatum* CECT 7765 supplementation significantly reduced interferon gamma levels in the intestine, both after early life stress and in non-stressed control mice. At PND41, approximately three weeks after cessation of probiotic treatment and early life stress, mice showed increased anxiety-like behaviour in the EPM, which was attenuated by probiotic treatment [98]. This suggests that concurrent probiotic treatment is sufficient to prevent emotional dysfunction induced by early life stress, though it was not tested whether probiotic treatment after early life stress could also prevent early life stress. On the whole, single-species probiotics have been shown to attenuate inflammation and anxiety-like behaviour in response to stress paradigms, although it remains unclear as to why a given probiotic strain is preferable over another.

The effects of multi-species probiotics have been less well investigated in rodents. The efficacy of eight bacterial strains (Bifidobacterium spp., Lactobacillus spp., Lactococcus spp. altogether) on affecting depression-like behaviour were assessed after HFD-induced obesity in rats [99]. Interestingly, probiotic intake was found to have a clear, independent effect on depressive-like behaviour in the FST. Total immobility time was significantly reduced in both lean and obese rats given the probiotic, whereas obesity with or without probiotic supplementation had no effect on depressive-like behaviour [99]. As expected, chronic HFD feeding elevated circulating levels of LPS, although in this case, probiotics had no effect. Plasma metabolomics was performed by mass spectrometry. HFD-induced obesity led to many (209) metabolite changes compared to lean rats, including a highly significant increase in the tryptophan catabolite and NMDA agonist [103] quinolinic acid. Following FDR correction, probiotic treatment increased the levels of two tryptophan metabolites, indole-propionic acid and indole-acrylic acid, as well as acetylornithine. While no significant diet x probiotic treatment interactions were found, indole-propionic acid has been shown to exert anti-inflammatory effects, attenuating astrocytic CCL2 cytokine expression in a mouse model of experimental autoimmune encephalitis [104]. By exerting this central anti-inflammatory effect, indole-propionic acid may contribute to the antidepressant-like effects of the probiotic mixture.

In the above studies, only male rodents were used as test subjects. While the studies discussed here are not an exhaustive review of the current literature, the vast majority have reported the effects on males only. Further studies exploring the possible interaction between probiotic treatment and sex, in healthy subjects and in disease models, are urgently required to identify, validate, and translate the therapeutic effects of microbiota modulation in mood disorders.

### 5.3. Prebiotics and Probiotics in Human Studies

The psychotropic effects of probiotics, and to a lesser extent prebiotics, have been assessed in humans, although the composition, timeframe, and nature of the human subjects varies greatly between studies (Table 2). In healthy subjects, a GOS prebiotic has been shown to significantly reduce the waking cortisol response after three weeks of treatment [105]. This effect was associated with reduced attentional vigilance to negative stimuli and increased vigilance to positive stimuli, which was significantly different from the placebo. Whilst this suggests some possibly anxiolytic effects of the prebiotic at a subconscious level, there were no significant changes in self-reported measures of anxiety or negative affect pre- and post-treatment. One possible reason for this was because the subjects were not clinically anxious or depressed at baseline. In fact, previous history of a psychiatric disorder was an exclusion criterion of the study [105]. Another study stratified healthy individuals into high and low trait anxiety at recruitment [106] and found that four weeks GOS treatment reduced self-reported anxiety scores in highly anxious participants, but not in individuals who had low levels of anxiety to begin with. This substantiates the notion that modifying the microbiota to improve mood may be most efficacious in people with mood disorders.

In people with irritable bowel syndrome (IBS), where anxiety is highly comorbid [107], GOS prebiotic treatment both increased levels of faecal Bifidobacteria and reduced anxiety scores using the Hospital Anxiety and Depression (HAD) scale at baseline and follow-up [108]. Despite some evidence pointing to greater efficacy of prebiotics in people with greater symptom severity, the one study that has tested (GOS) prebiotic treatment in people with MDD found no significant effect on Beck Depression Inventory (BDI) scores after an eight-week treatment regime [109]. The study power was comparable with other studies (n = 36–38 per group). Anxiety scores were not measured. Notably, about 10% of individuals taking the prebiotic experienced mild adverse effects including gastrointestinal complaints. Overall, there is limited evidence for the efficacy of prebiotics for improving mood, which is supported by a recent meta-analysis [110]. The evidence is especially sparce in people with clinically significant levels of anxiety and depression, and further studies are needed to determine whether prebiotics are effective in people with clinically significant anxiety disorders.

Probiotics have thus far been more extensively tested as a potential therapeutic for low mood and MDD. In healthy subjects, 30-day probiotic treatment with *Lactobacillus helveticus* R0052 and *Bifidobacterium longum* R0175 reduced anxiety and depressive symptoms using the HAD scale [111]. Conversely, BDI and Beck Anxiety Inventory (BAI) scores were not significantly different between pre- and post-treatment. *Lactobacillus rhamnosus* (JB-1), which showed promising preclinical results in reducing basal levels of anxiety-like behaviour in healthy mice [35], failed to improve mood or reduce anxiety in healthy volunteers after eight weeks of treatment [112].

Probiotics have also been trialled in individuals with subclinical levels of anxiety and depression. In individuals with mild to moderate levels of depression (Patient Health Questionnaire [PHQ]9 score 5–19), a four-week treatment with a multi-species probiotic (Bio-Kult Advanced^®^) significantly reduced PHQ9 scores, suggesting an antidepressant effect [113]. In contrast with previous research in prebiotics [105], salivary cortisol levels were unaffected by probiotic treatment, and no changes in blood C-reactive protein (CRP) levels were identified [113]. In individuals with moderate to severe depression, a probiotic mixture of *Lactobacillus helveticus* R0052 and *Bifidobacterium longum* R0175 did not improve psychological outcomes after eight weeks of treatment, including depression or anxiety [114]. The sample size was greater and the duration of treatment longer than a previously successful trial of the same probiotic mixture in healthy subjects [111].

Lastly, probiotics have been trialled in individuals with a clinical diagnosis of MDD. In the two studies that have sought to assess the antidepressant efficacy of probiotics for clinically significant depression, both showed an improvement in BDI score at follow-up with the probiotics compared to those given the placebo [109,115]. In both studies, both placebo and probiotic groups were receiving SSRI treatment—the use of the probiotic was therefore an adjunct therapeutic. One study trialled *Lactobacillus helveticus* R0052 and *Bifidobacterium longum* R0175 for eight weeks [109] and the other trialled *Lactobacillus acidophilus, Lactobacillus casei*, and *Bifidobacterium bifidum* for eight weeks [115]. In both cases, circulating inflammatory markers such as CRP and the kynurenine/tryptophan ratio were downregulated after probiotic supplementation compared to placebo.

**Table 2:** Summary of probiotic studies on anxiety and/or depressive symptoms in humans. ↑ increase, ↓ decrease.

Paradigm	Probiotic (Dose)	Duration of Treatment	Effects	Reference
Adult males and females (healthy)	*L. helveticus R0052* and *B. longum R0175* (3 × 10^9^ CFU, daily)	30 days	↓ anxiety and depression HAD scores No effect on BDI or BAI scores	[111]
Adult males (healthy)	*L. rhamnosus JB-1* (1 × 10^9^ CFU, daily)	4 weeks	No differences between probiotic and placebo for BDI, BDAI, or serum cytokine levels	[112]
Adult males and females (mild to moderate depression: PHQ9 score 5–19)	*Bacillus subtilis, Bifidobacterium bifidum, Bifidobacterium breve, Bifidobacterium infantis, Bifidobacterium longum, Lactobacillus acidophilus, Lactobacillus delbrueckii* ssp. *Bulgaricus, Lactobacillus casei, Lactobacillus plantarum, Lactobacillus rhamnosus, Lactobacillus helveticus, Lactobacillus salivarius, Lactococcus lactis* ssp. *Lactis, Streptococcus thermophilus.* (2 × 10^9^ CFU, daily)	4 weeks	No effect of probiotic on salivary cortisol or serum CRP Probiotic ↓ PHQ9 scores	[113]
Adult males and females (moderate or severe depression: ≥11 QIDS-SR16 or ≥14 DASS-42)	*L. helveticus R0052* and *B. longum R0175* (3 × 10^9^ CFU, daily)	8 weeks	No effects of probiotic treatment on self-reported depression or anxiety scales, and no effect on serum CRP, IL-1B, IL-6, TNF, BDNF, or vitamin D	[114]
Adult males and females (MDD diagnosis, moderate-severe symptoms)	*L. acidophilus*, *L. casei*, and *B. bifidum* (6 × 10^9^ CFU, daily)	8 weeks	Probiotic ↓ BDI score compared to placebo Probiotic ↓ serum CRP level compared to placebo	[115]
Adult males and females (MDD diagnosis, mild-moderate symptoms)	*L. helveticus R0052* and *B. longum R0175* (1 × 10^10^ CFU, daily)	8 weeks	Probiotic ↓ BDI score compared to placebo Probiotic ↓ serum kynurenine/tryptophan ratio compared to placebo	[109]
Adult pregnant women (at least mild anxiety or depression: ≥10 EPDS or ≥40 STAI)	*B. bifidum* W23, *B. lactis* W52, *L. acidophilus* W37, *L. brevis* W63, *L. casei* W56, *L. salivarius* W24, *Lc. Lactis* W19, *Lc. Lactis* W58 (5 × 10^9^ CFU, daily)	From 26 to 30 weeks gestation until delivery	No effect of probiotic on perinatal maternal depression (EPDS) or anxiety (STAI) scores	[116]
Adult pregnant women (healthy)	*L. rhamnosus HN001* (6 × 10^9^ CFU, daily)	From 14 to 16 weeks gestation until 6 months postpartum	Probiotic significantly ↓ depression (EPDS) and anxiety (STAI) scores compared to placebo	[117]
Adult pregnant women (obese: BMI ≥ 30 kg/m^2^)	*L. rhamnosus GG* and *B. lactis BB12* (6.5 × 10^9^ CFU, daily)	From 12 to 18 weeks gestation until 36 weeks gestation	No effect of probiotic on depression (EPDS) or anxiety (STAI-6) scores compared to placebo	[118]

### 5.4. Prebiotics and Probiotics during the Perinatal Period

Preclinical studies have investigated the effects of prebiotic and probiotic treatments during the perinatal period on offspring health outcomes, whereas clinical studies are lacking. There is currently no evidence for the consumption of prebiotics on maternal wellbeing outcomes—a recent systematic review did not identify any relevant studies using prebiotics [119]. A feature of recent and ongoing clinical trials is the effect on pregnancy outcomes [120] and the prevention of autoimmune-related disease in infants with conflicting results of both beneficial and no effects reported [121,122,123,124].

Maternal prebiotic intake has been shown to alter the behaviour of juvenile and adult offspring in mice [125]. Specifically, maternal GOS intake increased exploratory behaviour in young offspring compared to dams fed normal drinking water only. In adult offspring, anxiety-like behaviour was reduced and social behaviour was increased by maternal GOS intake. Age-dependent changes in NMDA subunit receptor expression was noted alongside increases in offspring faecal butyrate and propionate [125]. Direct stimulation of early life gut microbes through neonatal prebiotic administration from PND3 to PND21 has also been shown to increase NMDA receptor subunit GluN2A, synaptophysin, and brain-derived neurotrophic factor protein levels in the hippocampus at PND22 [126]. These effects persisted from weaning into early adulthood, at 8 weeks of age, despite the cessation of prebiotic supplementation. While gut microbiota analysis was not performed in this study, the same galacto-oligosaccharide prebiotic has been shown to be Bifidogenic in adult rodents [85].

A meta-analysis on the effect of probiotics on maternal metabolic health indicates that probiotic interventions improve glucose and lipid metabolism, including reducing insulin resistance [127]. Recently, probiotics have been investigated in clinical trials for their effects on maternal mental health during pregnancy and the postpartum period [119]. Completed clinical studies have shown mixed results. A 2021 study aimed to assess the effect of prenatal probiotic (n = 20) or placebo (n = 20) consumption from mid- to late gestation until delivery on maternal depression and anxiety symptoms during pregnancy [116]. Both Edinburgh Postnatal Depression Scale and State-Trait Anxiety Inventory scores decreased between the baseline and the eight-week follow-up in both probiotic and placebo groups. Thus, while there was a significant effect of time, there was no significant difference between the intervention and control groups. No biomarkers of treatment response were used, and faecal microbiome sequencing was not performed. A study of 380 women who received either *Lactobacillus rhamnosus* HN001 (193) or placebo (187) from 14 to 16 weeks’ gestation until six months postpartum were assessed for anxiety and depressive symptoms during pregnancy and the postpartum period [117]. This study found a reduction in these symptoms in the probiotic group both during and after pregnancy compared to those given a placebo. The large sample size and long duration of treatment of this study is a strength that should be replicated in future studies, particularly those that will assess offspring development. On the other hand, supplementation of *Lactobacillus rhamnosus* GG and Bifidobacterium lactis BB12, at a similar dose, did not affect depression and anxiety scores in an obese (BMI > 30.0 kg/m^2^) cohort of 164 women [118]. Probiotics were taken starting from a similar timepoint, from mid-gestation until birth rather than post-weaning. Hence, this was an assessment of mental wellbeing during pregnancy only, rather than postpartum anxiety and depression.

The effects of maternal probiotic consumption on offspring health outcomes have also thus far only been investigated preclinically, and the scope of research here is limited. Mouse prenatal probiotic supplementation has been suggested to increase serum IgG and intestinal secretory IgA [128] in the adult offspring, which may have immunoprotective roles [129]. In line with this, antibody responses to hepatitis-B surface antigen were shown to be higher in offspring from BGOS-supplemented dams compared to controls [128]. Similarly, supplementation of rat dams with Lactobacillus fermentum CECT5716 during pregnancy and the first two weeks of lactation increased IgA in offspring pups at postnatal day 14 [130]. *L. fermentum* supplementation also had a positive effect on plasma fatty acid profiles in both dams and offspring. The same experiment demonstrated that these effects may have been mediated by changes to the mammary milk composition, which showed increased polyunsaturated fatty acid contents, reduced palmitic acid, and increased IgA in probiotic-supplemented dams compared to controls [131]. Despite these changes, *L. fermentum* CECT5716 was only detected in half of the supplemented dams (3/6) suggesting that the presence of the probiotic in the milk was not a prerequisite for imparting positive immune effects on the offspring. Rather, changes to the intestinal microbiota, with subsequent systemic effects, may have mediated the beneficial effects on the rat milk composition [131].

Our laboratory has shown that faecal SCFA levels, specifically butyrate and propionate, are elevated in adult offspring of dams supplemented with a multispecies probiotic [132]. Maternal probiotic intake was also associated with reduced anxiety and depressive-like behaviour in these offspring, and faecal butyrate levels correlated positively with brain mRNA levels of PFKFB3, a marker of astrocytic metabolic activity. This suggests that early life exposure to the probiotic, possibly by maternal coprophagia or simply cohabitation, allows for long-term colonisation of the probiotic species that promote increased butyrate and propionate production and altered brain energetics. Likewise, another study [133] explored the effects of maternal CD, HFD, and HFD/probiotic during gestation and nursing in male rat offspring at 16 weeks of age (eight animals per group). In support of the results of our laboratory, a non-significant trend towards increased faecal butyrate levels was observed in the offspring of HFD/probiotic dams compared to CD dams (22% increase) and HFD dams (35% increase).

Overall, there is some evidence for a beneficial role of early life exposure, prenatal and postnatal, on maternal intake of prebiotics and/or probiotics to support normal brain and immune development. Further research in this area should be encouraged, particularly as to whether supplementation during pregnancy and lactation mitigates the consequences of maternal obesity on offspring neurobiology and behaviour [134]. This is because there is some evidence that offspring microbial reconstitution has been suggested to reverse neurological and behavioural deficits induced by a maternal high-fat diet [135], while other work suggests that microbial reconstitution post-weaning is not sufficient to rescue neurological adaptations that arise during development [25]. Studies that include maternal diet are also needed because the type of diet consumed by the host, and the subsequent metabolic and inflammatory profiles associated with the diet, may significantly impact the efficacy of probiotic administration in terms of its anti-inflammatory properties and ability to modify the brain and behaviour [136].

## 6. Gastrointestinal Engraftment of Probiotic Supplements

In humans, conflicting evidence exists as to whether probiotic supplementation exerts a lasting effect on gut microbiota composition. Supplementation of adults with *Bifidobacterium infantis* 35624 for eight weeks increased *B. infantis* 35624 in faeces compared to placebo; however, the levels of the probiotic declined to baseline levels at the follow-up timepoint, two weeks after ceasing the daily probiotic treatment [137]. In another study, *Bifidobacterium* spp. have been shown to colonise the adult human gut only transiently, for eight days [138]. Conversely, supplementation of healthy individuals with *Bifidobacterium longum* AH1206 remained detectable in stool for up to six months after discontinuation of the probiotic [139]. Human studies investigating the effect of maternal perinatal probiotic supplementation on the offspring faecal microbiota have also exhibited conflicting results. One study investigated the effect of a Lactobacillus-containing probiotic supplement, between the last month of gestation and the first 3 months of lactation, on the longitudinal microbiota of the infants. Sequencing of 16S rRNA revealed that the infants had increased levels of faecal *Lactobacillus rhamnosus* GG (contained in the probiotic) relative to control infants at 3 months of age, but not at 12 months [140]. However, in mothers supplemented with *Lactobacillus rhamnosus* GG during late pregnancy (but not lactation), children have been shown to have detectable faecal levels of *L. rhamnosus* GG until at least 12 months of age [141]. The infants of mothers who did not receive the probiotic had no detectable levels of *L. rhamnosus* GG at this timepoint.

There are two somewhat opposing mechanisms to explain why probiotics persistently colonise the gut in some individuals but not others. On the one hand, colonisation success may depend on the pre-existing level of conspecific strains in the gut. A 2016 study found that only probiotic strains of species already existing in the gut were able to be detected three months after probiotic treatment, the reason being that the species will already have the appropriate microenvironment with which to colonise successfully [142]. On the other hand, low levels of *B. longum* predict *B. longum* AH1206 engraftment success [139]. Further metagenomic analysis suggested that colonisation success was related to the under-exploitation of certain carbohydrates produced in the gut, providing a window of opportunity for the probiotic strain to establish itself.

While an indigenous “mature” microbiome is generally quite resistant to colonisation by probiotics, a microbe-deficient GF gut is readily colonised by probiotic species [143]. Accordingly, the neonatal gut may be much more readily colonisable with probiotic species, especially if they are obtained from the maternal milk or faecal matter. In humans, bacterial strains that colonise the infant gut in the first six months of life have been shown to persist for at least the next six years, even if they are not the dominant strain of a given species [144]. Similarly, multi-strain probiotic administration to pre-term infants resulted in the increased abundance of probiotic *Bifidobacterium* spp. strains up to five months later [145]. This is evidence for the notion that early engraftment may be an important factor in the longevity of colonisation and may contribute to early life prebiotic and probiotic interventions may be more efficacious than in adulthood.

## 7. Depression and Obesity: A Bidirectional Relationship

Depression and obesity are both common, multifactorial disorders that impart severe global health and economic burdens [4,146]. At an individual level, they can reduce the quality of life of those affected. Substantial epidemiological evidence exists for a bidirectional relationship between depression and obesity [147,148,149,150]. A meta-analysis of longitudinal studies found that obese individuals had a 55% increased risk of developing depression over time, while depressed persons were 58% more likely to become obese [147]. Reciprocally, weight loss has been shown to improve mood in people with depressive symptoms [151]. While antidepressant medication usage can cause short-term weight gain, medication status is not thought to mediate the relationship between obesity and depression [152].

Obesity and depression share genetic risk factors. Genome-wide association studies (GWAS) have identified an overlap between the genetic risk for depression [153] and obesity [154] through single-nucleotide polymorphisms near or in *OLFM4* and *NEGR1*. The association between *NEGR1*, obesity, and depression has been validated in independent human studies that cumulatively exceed 1.5 million individuals [155,156,157,158,159,160]. Transcriptomic analysis has mapped *NEGR1* expression in the CNS predominately to the hypothalamus in both humans [160] and rodents [161]. *NEGR1−/−* mice exhibit reduced brain volume, as well as reduced relative hippocampal volume [162]. This is thought to be due to the fact that *NEGR1* is required for ongoing neurogenesis in the adult hippocampus in mice [163]. *NEGR1−/−* mice also demonstrate increased anxiety-like behaviour in the OFT and EPM, and increased depressive-like behaviour in the FST and tail suspension test compared to wildtype controls [163]. In the hypothalamus, *NEGR1* function may be related to the central regulation of feeding behaviours and energy balance [164].

**Figure 2:**
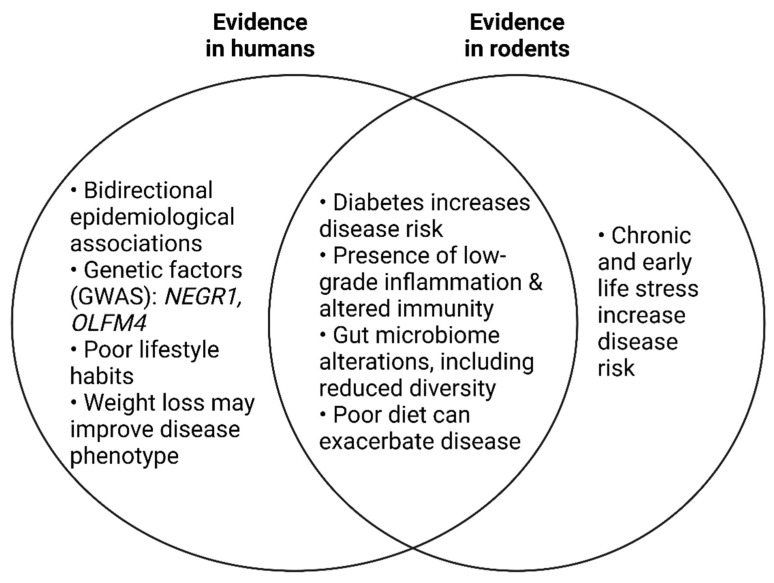
Summary of the evidence from human and/or rodent studies that demonstrate overlapping pathophysiology between obesity and major depressive disorder.

Chronic, low-grade inflammation is a hallmark of both obesity and depression [165] (Figure 2). In a large, case-control study of depression in the UK Biobank [38], low-grade inflammation was identified in 21% of patients (defined as serum CRP > 3 mg/L). In the same study, the genetic risk for lifetime depression was found to be positively correlated with serum CRP levels. Interestingly, while this signature of inflammation in depression was found to be independent of BMI and other clinical and demographic features, the correlation with genetic risk was entirely dependent on BMI and smoking status. BMI was higher in individuals with depression. Thus, it was concluded that the genetic contribution to an inflammatory phenotype in depression is entirely dependent on these two factors [38].

Limited preclinical evidence exists to support a role for the microbiota in the bidirectional relationship between obesity and depression. Transplanted gut microbiota from mice subject to HFD-induced obesity increased anxiety-like and stereotypic behaviour in recipient mice [166]. Recipient mice were first given broad-spectrum antibiotics to deplete the microbiome. Given that antibiotic treatment alone can alter behaviour [167], this may have confounded the results. Nevertheless, the obese-type gut microbiota were also found to increase intestinal permeability as well as peripheral and central inflammation without a significant change in body weight [166].

**Figure 3:**
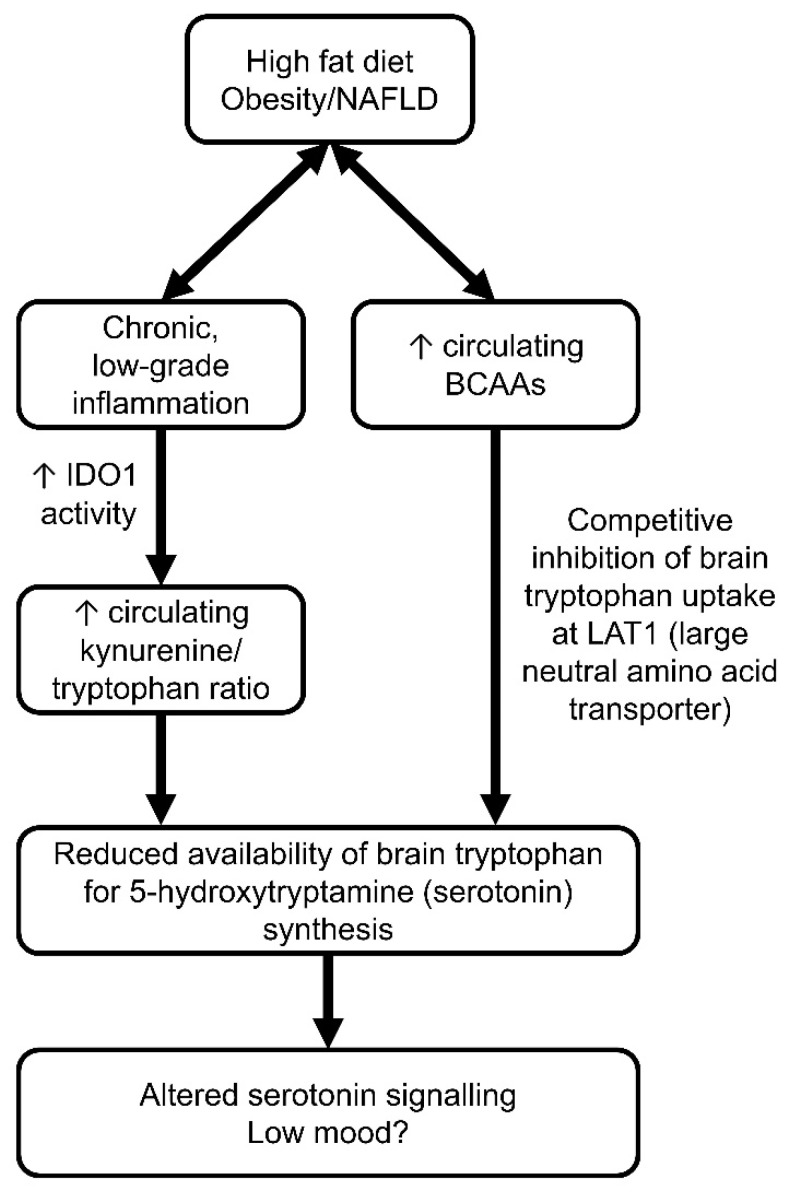
Potential mechanisms describing how diet-induced metabolic syndrome (obesity/NAFLD) may lead to altered brain plasticity and low mood. On the left side of the flowchart, chronic, low-grade inflammation is thought to upregulate peripheral indoleamine 2,3-dioxygenase (IDO1) activity, leading to increased kynurenine production from tryptophan and reduced brain tryptophan uptake for central serotonin synthesis. On the right, elevated circulating branched-chain amino acids may arise after prolonged high-fat diet feeding. BCAAs compete with tryptophan for the large neutral amino acid transporter (LAT1), reducing brain uptake of tryptophan and central serotonin synthesis. This may lead to altered neurotransmission and low mood. ↑ increase.

Isoleucine is a metabolite that may link depression and obesity pathophysiology. Increased isoleucine has previously been related to depression in both humans [168] and rodents [169,170]. Circulating isoleucine levels can affect the brain bioavailability of tryptophan, the key substrate for serotonin synthesis. Tryptophan and isoleucine (as well as other large, neutral amino acids such as leucine and valine) compete for uptake by the transporter LAT1 into the brain. Therefore, increased circulating isoleucine levels are thought to competitively reduce brain tryptophan uptake, thereby reducing brain serotonin levels and potentially altering behaviour [109,171] (Figure 3). Our laboratory has previously shown that, in an LPS model of post-inflammatory depressive-like behaviour (as assessed by the FST), increased plasma isoleucine levels also coincided with increased glutamine levels and reduced glutamate levels in the brain [170]. This may suggest that different models of depressive-like behaviour in rodents may converge on a similar mechanism involving altered circulating isoleucine and glutamate/glutamine ratios in the brain.

Increased isoleucine may arise due to an excessive level of dietary fat. Mice fed a HFD displayed elevated circulating BCAA levels alongside depressive-like behaviour and reduced extracellular hippocampal levels of serotonin [169]. In the same study, metformin reduced circulating BCAA levels and showed antidepressant-like effects in an HFD model of depression, suggesting that BCAA levels may indeed play a causal role in the pathophysiology of depression, especially in the context of metabolic dysfunction. Despite an apparent connection between diet-induced obesity and elevated circulating BCAAs, it is unlikely to be the diet itself that directly contributes to elevated BCAA levels. In the aforementioned study, BCAA levels were matched between the HFD and CD, whereby increased energy consumption was derived exclusively from the increased lipid content [169]. Rather than through diet, elevated fasting BCAAs are thought to be due to impaired catabolic pathways through the branched-chain keto acid and acyl-CoA pathways [172]. To date, no study has tested whether enzymes responsible for BCAA catabolism are altered in expression or activity in individuals with depression, so it remains unclear whether this mechanism—which likely underlies the association with elevated BCAAs in obesity [173]—may also account for the observation of elevated isoleucine in individuals with depression.

Overall, there is substantial epidemiological evidence for a bidirectional relationship between obesity and depression, which may be mediated by chronic low-grade inflammation or other genetic factors associated with the regulation of food intake. Isoleucine may be important to this concept, because existing evidence suggests that its elevation may be a consequence of altered metabolism in obesity [173] and a contributory cause of depression by depleting brain bioavailability of tryptophan. Studies in humans are required to determine the putative role for obese-type gut microbiota in peripheral inflammation and depression.

## 8. Conclusion and Perspectives for Future Research

The microbiota–gut–brain axis is mediated by a complex network of neural, endocrine, and immune signalling pathways, as well as by the release of various metabolites and neurotransmitters that can influence both gut function and brain function [1]. In both healthy [57] and diseased rodent models [37], the gut microbiota and associated metabolites have been demonstrated to regulate brain development, plasticity, and behaviour. In humans, dysfunction of the microbiota–gut–brain axis through obesity and metabolic disease has been implicated in a range of psychiatric and neurological disorders, including depression [83].

Prebiotics and probiotics, through their modulation of the gut microbiome, have emerged as potential therapeutic options for the management of depression [2]. While more research is needed to fully understand the complex mechanisms underlying this relationship, the evidence so far suggests that interventions targeting the gut microbiota have not been convincingly translated in clinical trials, though more studies are required in patients with moderate to severe symptoms. Ultimately, a comprehensive approach to mental health should take into account the bidirectional relationship between the gut and brain, and consider the potential role of prebiotics and probiotics in promoting optimal gut health and overall well-being.

Moreover, the use of prebiotics and probiotics is relatively safe and well-tolerated, making them an attractive option for individuals who are looking for natural and non-invasive interventions for depression. It is important to note, however, that not all prebiotics and probiotics are created equal, and their efficacy and safety may vary depending on the specific strain, dose, and formulation.

Moving forward, a key area of research in the field of prebiotics and probiotics for depression is to better understand the specific mechanisms by which these interventions modulate the gut microbiome and ultimately affect mental health outcomes. In terms of uncovering key molecular and metabolic intermediates in microbe-to-brain communication, isotopically labelled tracers (e.g., ^13^C-labelled glucose, acetate, or lactate) should be considered. Depending on the positioning of the labelling at each carbon atom, the kinetics of different metabolic pathways can be inferred following prebiotic and probiotic treatments [174].

Placebo-controlled clinical trials that explore the potential use of prebiotics and probiotics to target specific subtypes of depression, such as treatment-resistant depression or postpartum depression, are also needed. By shedding light on the precise mechanisms by which prebiotics and probiotics impact mental health, future research in this field has the potential to uncover novel therapeutic targets and ultimately lead to more effective treatments for depression.

## Data Availability

No new data were created or analyzed in this study. Data sharing is not applicable to this article.
